# Potential Food Inclination of Crab-Eating Macaques in Laboratory Environments: Enhancing Positive Reinforcement Training and Health Optimization

**DOI:** 10.3390/ani14071123

**Published:** 2024-04-07

**Authors:** Ji Woon Kim, Yoon Beom Lee, Yeon Su Hong, Hoesu Jung, Gwang-Hoon Lee

**Affiliations:** Preclinical Research Center, Daegu-Gyeongbuk Medical Innovation Foundation, Daegu 41061, Republic of Koreahong6x@kmedihub.re.kr (Y.S.H.); junghs2000@kmedihub.re.kr (H.J.)

**Keywords:** crab-eating macaque, positive reinforcement training, welfare, dietary choice, primate, behavioral assessment, health optimization

## Abstract

**Simple Summary:**

This study examined the potential food inclination of crab-eating macaques, a species that frequently participates in animal behavior research, in a laboratory environment. This study aimed to understand the potential food inclination of crab-eating macaques to enhance positive reinforcement training and health optimization, which are important for behavioral assessments. We observed a strong inclination for plant-based foods like fruits and vegetables over animal-based proteins. Notably, nuts were highly favored. Additionally, our research revealed individual variation in inclination and a time-dependent increase in protein inclination. Our results emphasize the need for a diverse range of inclined foods in positive reinforcement training and health optimization, with consideration for individual variation. This study offers guidelines for ethical and effective training practices, ensuring the well-being of these monkeys in research settings. This understanding of their potential food inclination can improve the overall welfare of crab-eating macaques, enhance the success of behavioral assessments, and provide valuable insights for researchers in various fields.

**Abstract:**

Positive reinforcement and training for health optimization are pivotal for successful studies with monkeys. Potential food inclination is important for studies on crab-eating macaques in laboratory environments, but evaluations remain scarce. We explored crab-eating macaques’ potential food inclination to establish a reward system for future behavioral assessments. Twelve male and three female monkeys underwent a food inclination assessment in which they were offered four food categories—fruits, vegetables, proteins, and nuts. The monkeys exhibited a higher inclination for plant-based foods, particularly fruits and vegetables, over animal-based proteins like chicken and tuna (*p* < 0.0001), with a notable inclination for nuts (eaten/provided = 100%). Additionally, the consistency of potential food inclination after repeated offerings was investigated, revealing a time-dependent increase in inclination for protein items. Food consumption ratios correlated positively with caloric intake (*r* = 0.59, *p* = 0.02), implying that individuals with a regular high caloric intake and increased body weight are more likely to accept food during positive reinforcement training. Our findings suggest fruits, vegetables, protein-rich foods, and nuts can help with health optimization. However, animal-based protein-rich foods initially had a low preference, which may increase over time. Our study can provide guidelines for positive reinforcement training and health optimization.

## 1. Introduction

Crab-eating macaques (cynomolgus monkey, *Macaca fascicularis*, long-tailed macaque) have long been considered one of the most widely distributed nonhuman primates (NHPs) [1]. Naturally found throughout Southeast Asia, this species inhabits diverse habitats, including forests and urban environments [2,3]. The monkeys have been extensively traded for scientific research and are recognized as an essential species in preclinical studies. In biomedical experiments, monkeys are vital subjects, often subjected to confinement and exposed to various interventions [4,5]. The crab-eating macaque, owing to its notable parallels to humans in terms of genetics, behavior, metabolism, anatomy, physiology, brain function, and endocrine system, serves as a valuable model for a diverse range of preclinical evaluations prior to clinical phase entry, including, but not limited to, drug delivery, toxicology, cardiovascular disease, and immune system development [6,7,8]. Additionally, this species possesses a brain structure that is highly analogous to that of humans and exhibits humanlike behavioral patterns, so it is frequently used in behavioral experiments, underscoring its utility as an advanced animal model [9,10]. Behavioral experiments refer to employing methods to understand and measure patterns of behavior, social interactions, learning abilities, and stress responses in monkeys.

The assessment of crab-eating macaque behavior is vital in the fields of ethology and psychology, which could enhance our understanding of higher intelligence and intricate social behavior. Such studies augment the value of these animals as experimental and research subjects and include, for example, assessments of female crab-eating macaques to determine behavioral and neurobiological characteristics that influence social hierarchy formation and model depression [11,12]. However, the evaluation and interpretation of complex behaviors often pose challenges. Therefore, understanding the rewards that trigger and reinforce behavior is pivotal [12,13]. To enhance the success rate of behavioral assessments, procedures involving negative background stimuli can be employed, such as water restriction or fasting followed by food reinforcement. However, such approaches may have adverse effects on the welfare of the captive NHPs [14]. Alternatively, the use of positive reinforcement in a normative environment to induce the desired behavior does not compromise the captive NHPs’ welfare [14,15].

Consequently, various positive reinforcement training procedures have been developed to improve the success of behavioral assessments while considering the NHPs’ welfare [13,15]. Such methods collectively utilize positive rewards to reinforce and promote desired behaviors. The core concept is that animal behaviors are more likely to occur when test subjects are rewarded upon achieving desired outcomes, thereby reinforcing the associated behavior [13,15,16]. Indeed, effective food reinforcement has been widely employed to successfully enhance the performance of animal experiments [14,16]. Positive reinforcement training can be applied not only in research settings but also in captive management. Initially, it can be used to entice and acclimate animals to the captive environment [13]. Furthermore, it is applicable in veterinary procedures such as during blood sampling, socialization, separation, and target training. Positive reinforcement training allows staff and managers to actively address various situations that significantly impact animal welfare [17,18].

Specific food reinforcement strategies have increased success rates in research endeavors in captive settings and have shown potential for enhancing animal welfare in limited laboratory environments [19,20,21]. Food preference studies have been conducted on various NHPs, including orangutans [16], spider monkeys (*Ateles* spp.) [22], capuchin monkeys (*Cebus apella*) [23], bonobo chimpanzees (*Pan paniscus*) [21], and rhesus macaques (*Macaca mulatta*) [12]. Huskisson et al. explored food preference in a preliminary behavioral study using a touchscreen. Considering our goal of conducting potential food inclination assessments during behavioral research, we deemed their study relevant [24]. Martin et al. used the multiple stimulus without replacement method as an approach for researching food item preference. The aim of their study resembles ours, focusing on enhancing animal performance in husbandry and research operations [12]. Additionally, Rehrig et al. reported on the potential food inclination of crab-eating macaques [25]. However, the study was limited to food preference assessments for only six items, thus not evaluating a large number of food items. To perform potential food inclination assessments, an extensive evaluation of numerous food items is necessary. The food preference study involved participants selecting their preferred food item among two or more options, making it impractical to evaluate a large number of food items. Instead, we opted to assess whether monkeys showed tendencies to eat or avoid certain foods through a potential food inclination test using a wider range of food items. Previous research has explored the role of social influences using novel foods in monkeys; however, to our knowledge, there has been no prior experimentation identical to the potential food inclination test [26,27,28]. The use of the potential food inclination test is necessary for laboratory monkeys for health optimization. Monkeys in laboratory settings face various challenges that threaten their health. These challenges can include restraint, surgeries, administration of candidate drugs, and stress in limited spaces, which can lead to loss of appetite and nutritional imbalance [29,30,31].

Primate diets are designed to achieve an ideal nutritional balance. However, if a monkey reduces or eliminates intake of the provided diet for any reason, then the loss of nutrients must be compensated for with alternative food sources. Offering foods that provide a balanced intake of carbohydrates, fats, and proteins is essential. This is particularly true following invasive procedures, such as surgeries, when it is important to sufficiently supplement protein sources [32]. Protein provides the energy needed for tissue regeneration and recovery [33]. Cynomolgus monkeys typically require a minimum of 15% protein in their diet, increasing to 20% for those that are lactating [34]. Sandhyamani et al. reported pancreatic degenerative lesions in monkeys that received diets deficient in protein, highlighting the importance of adequate protein intake [35]. However, the literature on the potential food inclination of crab-eating macaques is lacking. To our knowledge, there have been no studies on novel foods, nor have there been any studies on the potential food inclination test [36].

Conducting assessments requires an evaluation of a diverse array of food items. Therefore, the present study focused on crab-eating macaques as experimental animals and aimed to identify their potential inclination for diverse food items. Additionally, potential food inclination and the consistency of potential inclination were evaluated through multiple offerings, and the necessity for diverse food provision was assessed. Evaluating the stability of potential food inclinations is helpful for recognizing potential inaccuracies associated with preferences [37]. The findings of the present study could enhance our understanding and facilitate the improvement of experimental and husbandry practices for NHPs, in addition to the enhancement of the welfare of NHPs.

## 2. Materials and Methods

### 2.1. Animal Ethics Statement

The animal study protocol was approved by the Institutional Review Board of Daegu Gyeongbuk Medical Innovation Foundation (protocol code KMEDI-22111001 and approval date: 10 November 2022). Animals were maintained in a facility accredited AAALAC International (#001796) in accordance with Guide for the Care and Use of Laboratory Animals 8th edition, NRC (2010).

The present study also adhered to the principles of the American Society of Primatologists for the Ethical Treatment of Nonhuman Primates. All animal experiments were performed in accordance with the animal protection regulations in the Republic of Korea, and animals were maintained in accordance with the Guide for the Care and Use of Laboratory Animals, 8th edition. In accordance with the principles, regulations, and guides, the health of experimental animals was overseen by attending veterinarians, ensuring their well-being through regular health check-ups. Additionally, post-approval monitoring by the Institutional Animal Care and Use Committee (IACUC) was implemented to verify fundamental animal welfare, and routine inspections were conducted to ensure that experiments were progressing according to the approved IACUC protocol. Standard operating procedures were established for evacuating animals as a priority in the event of facility emergencies. For the safety of researchers and staff when handling animals, the use of personal protective equipment was mandated. Standard operating procedures were also developed for safely capturing monkeys in the event of an escape. Considering that monkeys can harbor zoonotic diseases, a quarantine system of at least 31 days was established to safeguard human health.

### 2.2. Study Animals

Twelve male and three female crab-eating macaques (cynomolgus monkey, *M. fascicularis*, long-tailed macaque) aged 2–3 years were purchased from Keyprime Research Co., Ltd. (Cheongju, Republic of Korea) and the Primate Resources Center, Korea Research Institute of Bioscience and BioTechnology (Jeongeup, Republic of Korea), respectively. The average weight of the male monkeys was 2.60 ± 0.17 kg, and that of the female monkeys was 2.56 ± 0.34 kg. The overall average weight was 2.59 ± 0.20 kg. They were raised in the Preclinical Research Center of the Daegu-Gyeongbuk Medical Innovation Foundation. Similar food preference studies on other NHPs by Martin et al. [12], which involved 24 rhesus monkeys, and by Huskisson et al. [24], which employed 10 female primates, including *Gorilla gorilla, Pan troglodytes,* and *Macaca fuscata*, served as references for our choice of sample size in the present study.

### 2.3. Animal Husbandry

The study animals were maintained in a facility accredited by the Association for the Assessment and Accreditation of Laboratory Animal Care International (#001796). Each monkey was provided with a daily diet comprising eight pellets of gamma-irradiated certified global primate diet containing 20% crude protein pellets (#2050; Envigo, Indianapolis, IN, USA), a quarter of an apple, half a banana, and three UV-disinfected cherry tomatoes. Water (purified via microfiltration and reverse osmosis) was made available ad libitum. The housing environment was maintained at a temperature of 22 ± 1 °C, humidity of 50% ± 10%, negative room pressure of 3 mm Aq, 12 h light–dark diurnal cycle, and a ventilation cycle of 10–20 occurrences per hour.

All monkeys were housed individually to assess their dietary choices in the experimental period because, under social housing with two or more individuals, hierarchical relationships among the monkeys led to potential monopolization of food items and the basic feed provided by a dominant individual. Social housing may influence potential food inclination by introducing a potential variation in hunger levels due to the monopolization of food by a single monkey, impacting the assessment of potential food inclination. However, outside the experimental period, social housing was provided for the welfare of the animals. Furthermore, after the introduction of individual housing, there was a two-week acclimation period before the subjects were included in the experiments.

In acknowledgment of the advanced perceptual abilities and natural behaviors of monkeys, various environmental enrichments and stress-alleviating methods were introduced. Each cage possessed dimensions of 650 (width) × 800 (depth) × 1600 (height) mm to provide a floor area of 0.52 m^2^, surpassing the international guideline of 0.4 m^2^ as established by the National Research Council of the USA [38]. The “Guide for the care and use of laboratory animals” published by the National Research Council recommends providing a floor area/animal of 0.28 m^2^ for animals up to 3 kg and 0.4 m^2^ for animals up to 10 kg. Additionally, when suspended from the cage ceiling, the tail or legs should not touch the floor. We ensured compliance with these guidelines by providing a height of 1600 mm [38]. All cages were furnished with mirrors and bars, accommodating the climbing behavior typical of NHPs in their natural habitats, and available enrichment items included toys (KONG, Golden, CO, USA) and sterilized stainless steel balls.

**Figure 1:**
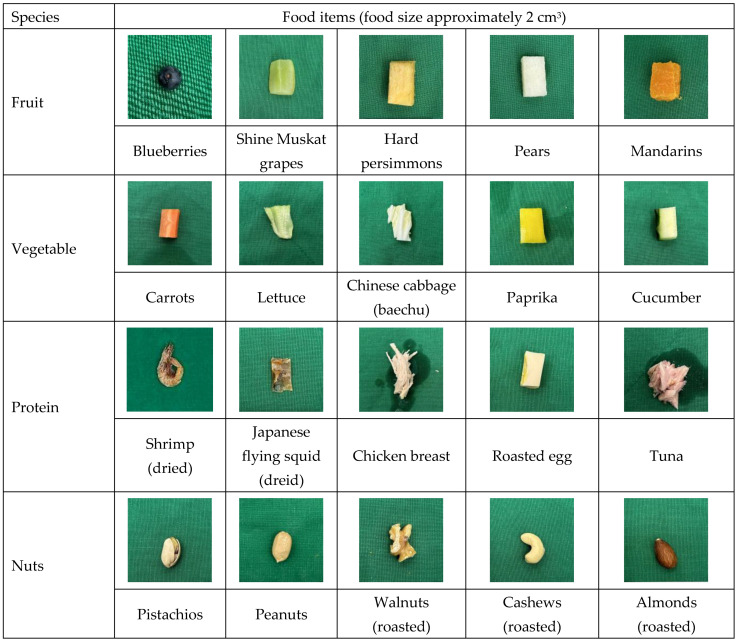
Photographic food items we presented to primates.

Food items were categorized into four groups: fruits, vegetables, protein items, and nuts. Each category contained five distinct food items (Table 1) for experimentation (supplementing the daily pellet feed, apples, bananas, and cherry tomatoes), namely: fruits—blueberries, Shine Muskat grapes, hard persimmons, pears, and mandarins; vegetables—carrots, lettuce, Chinese cabbage (baechu), paprika, and cucumbers; protein items—shrimp (dried), Japanese flying squid (dried), chicken breast, roasted egg, and tuna; nuts—pistachios, peanuts, walnuts (roasted), cashews (roasted), and almonds (roasted) (Figure 1, Table 1). In the present study, the criteria for selecting food items were based primarily on options that monkeys would naturally consume. However, considering the need for positive reinforcement training at the laboratory level, the researchers opted for foods that could be obtained and provided by the experimenters. In addition, raw eggs could not be standardized in size, and their consumption could not be ascertained reliably; therefore, they were excluded from the food items. The selected food items were selected to mimic those consumed naturally by monkeys as closely as possible. As the primary focus of our research was on the laboratory environment, any potential inclination for food was because it could be procured and readily provided in 2 cm^3^ portions.

The nutritional composition of each food item was obtained from the Food and Nutrition Ingredients Database provided by the Ministry of Food and Drug Safety [39] of the Republic of Korea.

### 2.4. Experimental Procedure

Our experimental protocol was built upon prior research methodologies [21], but it was adapted to minimize interference with daily dietary patterns by scheduling trials 3 h after the first daily meal and immediately preceding the second one. For the first meal, four primate diet pellets and a quarter of an apple were provided. For the second meal, four primate diet pellets, half a banana, and three cherry tomatoes were provided. The food items selected for experimentation were also novel to these primates, avoiding prior exposure to specific dietary elements.

To circumvent potential competition-induced biases in food selection, each primate underwent individualized trials. Each food item was presented once daily to each individual, and the presentation of all 20 food items was repeated five times during the trials. To standardize the food size and mitigate size-related effects, all food items were cubically sectioned to approximately 2 cm^3^ to minimize the effect on body weight. A previous study reported that the food provided for enrichment to the monkeys should not exceed 30 kcal/kg” [40,41]. In the present study, food items did not surpass approximately 3 kcal/kg of the regular daily diet. Furthermore, 2 cm^3^ was determined to represent the appropriate size that monkeys can grasp.

Initially, the observer presented each food item to the monkeys manually, allowing a visual check by the monkeys for 5 s. Subsequently, trial food items were offered to the monkeys in a feeding trough with dimensions of 10 (width) × 5 (depth) × 10 (height) cm. The subjects were observed for 10 min to ascertain whether they grasped and consumed the presented food item; if feeding did not commence within 10 min, the food items were removed. To avoid observer bias as a confounding factor, only a single researcher conducted all the food trials. After placing the food item in the feeding trough, the researcher exited the housing facility and only returned after 10 min to complete the observation, thereby minimizing the possible bias induced by the experimenter [11]. A 10 min interval was considered a sufficient duration for assessing an animal’s reinforcement preferences. While the monkeys could consume the food immediately or taste it and potentially spit it out, the time used to place it in the cheek pouch and either swallow or spit it out did not exceed 10 min. As visual confirmation, including placement in the cheek pouch through cameras, was obstructed by cage structures, a direct visual inspection was performed after 10 min to ensure accurate observations. Additionally, according to previous studies, food items were presented randomly in each trial to minimize the potential impact of previous food item presentations [12].

### 2.5. Analysis of Potential Food Inclination

A total of 1500 trials were conducted, consisting of 100 attempts (5 repetitions × 20 food items) per individual (*n* = 15). To mitigate variability, only a single observer was used to systematically assign scores. The scoring system comprised a score of 1 if the primate fully consumed the provided food item and a score of 0 if the food item remained uneaten or if only a small portion was consumed and the remainder was discarded. This methodology also ensured consistency in the evaluations across all trials.

### 2.6. Comparison of Weight Gain and Food Item Eaten/Provided Rates

The weight gain of subjects was compared with the rates of eaten/provided food items. Weight measurements were taken both before and after the completion of 100 d of potential food inclination experiments. This evaluation assessed whether a discernible association exists between the weight gain ratio and consumption rates of the food items provided. Weight measurement was conducted by placing the monkey in a portable cage designed for weight measurement. The total weight of the portable cage with the monkey was measured, and then the weight of the empty cage was subtracted to obtain the measurement. Since the monkeys were trained and voluntarily entered the portable cage, stress was minimized.

### 2.7. Comparison of Total Calorie Intake and Food Item Eaten/Provided Rates

Each monkey was provided with a daily diet comprising eight pellets of gamma-irradiated certified global eight primate diet containing 20.0% crude protein pellets, a quarter of an apple, half a banana, and three UV-disinfected cherry tomatoes. The residue of the provided food was checked the following morning, and caloric intake was calculated accordingly.

### 2.8. Statistical Analysis

All statistical tests were performed using the statistical software RStudio 2023.06.0+421 (RStudio Inc., Boston, MA, USA). Categorical and nominal variables were compared using chi-squared tests with Bonferroni correction. Chi-squared tests for overall homogeneity were run prior to further comparisons. Subgroup analysis of nominal variables was conducted using the chi square or Fisher’s exact test with Bonferroni correction. Chi-squared test for categorical variables with an expected count of at least 5, and Fischer’s exact test for categorical variables with expected count of less than 5. A Bonferroni correction was applied for pairwise comparison. All adjusted *p*-values < 0.05 were considered statistically significant.

## 3. Results

### 3.1. Consumption Ratios per Food Item Category

**Figure 2:**
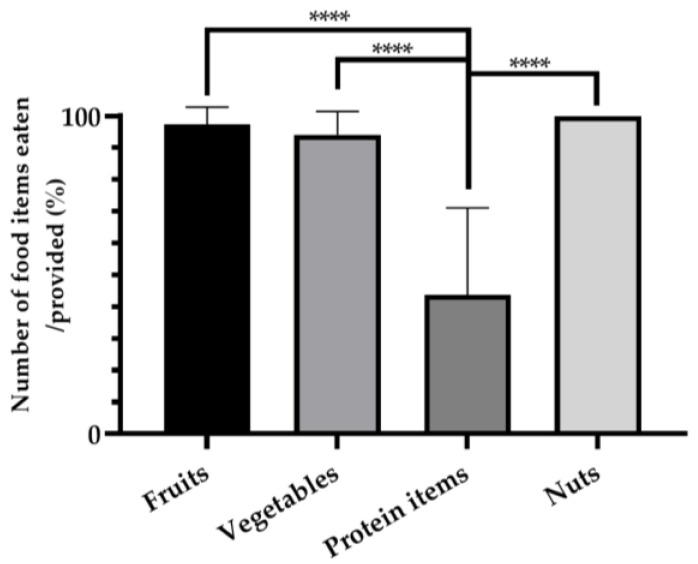
Consumption ratios per food item category (**** *p* < 0.0001 vs. protein items).

Consumption ratios were calculated as the number of food items ingested as a percentage of the total number of those specific items being offered to the subject. The crab-eating macaque subjects exhibited a mean consumption ratio of 97.33 ± 5.44% when fruits were presented, 94.13% ± 7.28% for vegetables, 43.73% ± 28.10% for protein items, and 100% ± 0.00% for nuts. Notably, the consumption ratio of protein items was significantly lower than that of the other categories (Figure 2).

### 3.2. Variations in Food Consumption across Trials

**Figure 3:**
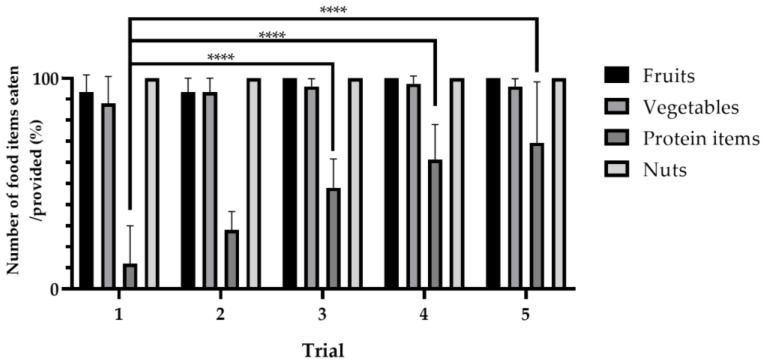
Variations in food consumption across trials (**** *p* < 0.0001 vs. protein items).

The consumption ratios of fruits, vegetables, and nuts ranged from 86% to 100%, with no statistically significant differences across trials. In contrast, the consumption of protein items increased incrementally as trials progressed, from 12.00% ± 16.60% (first trial) to 28.00% ± 8.69% (second trial) to 48.00% ± 13.66% (third trial) to 61.33% ± 23.76% (fourth trial), and finally 69.33% ± 28.90% (fifth trial). Notably, the rates recorded for trials 3, 4, and 5 were significantly higher than those of the first trial (Figure 3).

### 3.3. Individual Consumption Ratio of Protein Items across Trials

**Figure 4:**
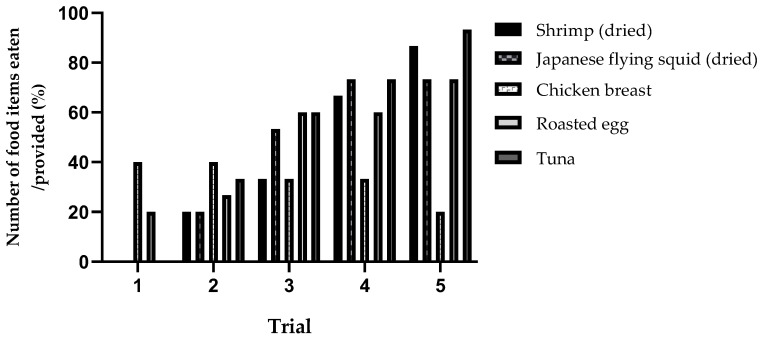
Individual consumption ratio of protein items across trials.

As the trials were repeated, the consumption ratios increased for dried shrimp, dried Japanese flying squid, roasted egg, and tuna, while they decreased for chicken breast (Figure 4).

### 3.4. Comparison of Weight Gain and Food Item Consumption Ratios

**Figure 5:**
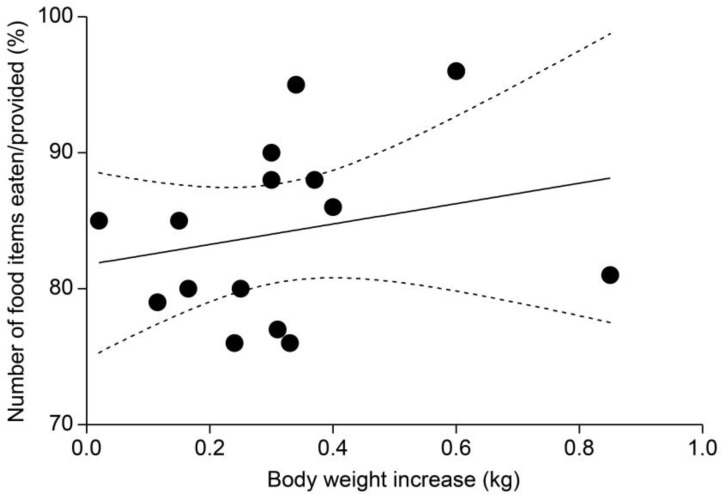
Correlation between subject weight gain and food consumption ratios (*r* = 0.23, *p* = 0.33).

A positive correlation was identified between weight gain in subjects and their consumption ratio of food items (*r* = 0.23). However, this relationship was not statistically significant (*p* = 0.33) (Figure 5).

### 3.5. Comparison of Total Caloric Consumption and Food Item Consumption Ratios

**Figure 6:**
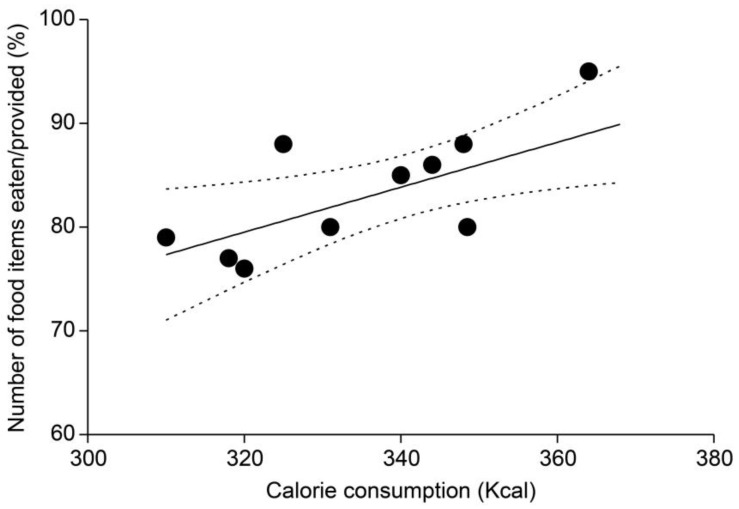
Correlation between total caloric consumption and the consumption ratios (*r* = 0.5901, *p* = 0.021).

A significant positive correlation was observed between total caloric consumption and the consumption of daily food items, (*r* = 0.5901, *p* = 0.021) (Figure 6). The total caloric consumption of male monkeys was 341.96 ± 18.96 Kcal, and that of female monkeys was 338.67 ± 13.05 Kcal. The overall total caloric consumption was 341.30 ± 17.57 Kcal. The difference in the mean total caloric consumption between males and females was small (3.29 Kcal).

## 4. Discussion

This study investigated the dietary choices of crab-eating macaques when presented with different food items from fruit, the vegetable, protein item, and nut categories. Their preferences were assessed after being offered 20 different food items (five times each). The results revealed a higher preference for plant-based foods and nuts than for animal-based protein-rich foods. Although the initial preference for various protein items was relatively low, an interesting temporal trend emerged, indicating an increase in the protein eaten/provided ratio over time.

Compared to other experimental animals, NHPs exhibit notably high intelligence and possess fine motor skills that are similar to those of humans, making them frequently used subjects in behavioral assessment studies [11,13,14]. In addition, the implementation of an appropriate positive food reward system enhances the success rate of such behavioral assessments while also reducing stress in NHPs. Martin et al. [12] reported that an assessment of NHP potential food inclination prior to behavioral evaluations resulted in increased participation during training sessions. Additionally, NHPs should be provided with a variety of preferred foods as part of their environmental enrichment, rather than being offered only standard diets [20,42,43].

*Macaca* species are omnivorous, prefer plant-based diets, and consume a wide range of items, including fruits, leaves, flowers, shoots, roots, insects, and small animals [44,45,46]. However, the proportion of fruits and vegetables in their diet tends to be higher than that of other food types [47]. With respect to fruit, macaques consume various fruits, including aromatic and non-aromatic fruits, protected and unprotected fruits, and fruits of all colors [48]. Our results revealed that crab-eating macaques exhibit a higher eaten/provided ratio for plant-based foods (such as fruits and vegetables), while animal-based foods rich in protein (such as chicken and tuna) elicit a lower eaten/provided ratio. This finding agrees with that of a previous study, which reported that the natural diet of wild crab-eating macaques mainly consisted of fruits or vegetables, with only approximately 1% meat [49]. In their natural habitat, monkeys supplement their protein intake with insects and earthworms, and approximately 7–14% of the primate diet consists of proteins to ensure sufficient consumption [49,50]. A fundamental principle in primate research is that no other living organism can be introduced into the laboratory as it may pose a risk of microbial contamination that could compromise the health of the primates. The unknown microbial status of any introduced organism necessitates this precaution. Protein is an essential component of the primate diet, necessary for its survival [51]. A decrease in dietary intake at the laboratory level can lead to nutritional imbalances. During the management of laboratory monkeys, reduced primate diet intake may occur after surgery, injection of new candidate drugs, or confinement in small spaces, meaning that the primates will not meet their nutritional needs. In such instances, a protein source that helps maintain muscle mass should be introduced over high-sugar foods like fruits, which are dominant in carbohydrates. Therefore, it is necessary to prepare non-living protein sources in addition to feed in the laboratory environment.

According to our research, crab-eating macaques initially showed a considerably lower consumption/provision ratio for protein items compared to fruits and vegetables, but this ratio increased over time. Therefore, it is suggested to continue offering, not discontinue, proteins if the monkeys do not initially consume them. Continuous trials of providing protein should be conducted. Our research results initially showed a low potential food inclination for protein items, but it gradually increased over time. Furthermore, it is noteworthy that out of the five food items tested, the potential food inclination increased over time for four items (dried shrimp, dried Japanese flying squid, toasted egg, and tuna), while it decreased for chicken breast. Based on our findings, we suggest that shrimp, Japanese flying squid, roasted egg, and tuna should be provided to laboratory monkeys for positive reinforcement training and health optimization, while chicken breast should not be provided. Contrary to wild primates, which maintain a consistent protein intake in their natural habitat, laboratory primates rely solely on food provided by humans, necessitating the provision of a consistent protein supply.

Our findings also demonstrated individual variation in potential food inclination among the subjects, with eaten/provided ratios in the 76–96% range. This underscores the nuanced nature of gustatory inclinations and emphasizes the importance of acknowledging individual variability in response to dietary offerings. These individual responses are crucial to consider during positive reinforcement training and health optimization to achieve successful outcomes. In addition, it is important to establish whether potential food inclinations remain stable over time. In our study, the same food item was provided five times to each subject to assess consistency in the behavioral response. The results indicated a change in some potential food inclinations over time, suggesting that some potential food inclinations can be adapted. While the monkeys maintained a consistently high eaten/provided ratio for fruits, vegetables, and nuts, their preference for protein items exhibited a time-dependent increase. During positive reinforcement training, the introduction of a variety of preferred foods is therefore preferable, rather than solely offering non-preferred items. This approach contributes to the successful implementation of positive reinforcement training and health optimization.

Lastly, food consumption patterns were positively correlated with caloric intake. Individuals that routinely consume a relatively large amount of feed and food are deemed slightly more likely to consume a greater number of new food items when introduced. Nevertheless, positive reinforcement training should not be solely based on offering preferred foods; it should also consider factors such as body weight and dietary habits. Additionally, food consumption patterns exhibited a positive correlation with weight gain. Increased caloric intake and weight gain can occur solely through the consumption of feed by primates. However, curiosity towards new foods and inclination may differ from feed intake alone. Hence, an evaluation was necessary to assess whether primates would consume newly introduced foods in addition to feed. The positive correlation with food consumption patterns suggests that inclination towards new foods may also be related to habitual calorie intake and weight gain.

Our study had some limitations, such as the absence of food-related foraging activities. In natural habitats, NHPs engage in foraging, which influences their food selection. Their natural behavior must be respected in the laboratory, necessitating the simulation of an environment where the NHPs can forage, which would contribute to their dietary habits and enhance their overall welfare. Another limitation is that potential food inclination may shift when items are offered more than five times, as has been observed in a previous study [52]. Another study involving an extended duration to investigate potential food inclination and measuring cortisol levels in fur is essential to exploring the relationship between stress and potential food inclination. Furthermore, single housing may have induced stress in monkeys. Real-time monitoring via internet protocol cameras can provide brief periods of social housing after all provided food items are consumed, which may contribute to their welfare improvement.

Dietary adaptation in monkeys may reveal insights into the interactions between humans and primates. Another limitation of the present study was the absence of a food preference assessment, which involves comparing two different foods to determine preference. In a future study, evaluating food preferences by comparing the 20 food items used in the present study would provide insightful results. Additionally, the present study exclusively involved immature crab-eating macaques that had not achieved sexual maturity. The male-biased sex ratio in our study presents a limitation in generalizing results to the entire crab-eating macaque population. To address the limitation, similar experiments should be conducted with female cohorts, ensuring visual and olfactory separation of males and females in the housing facility. Given our study’s provision of a small size (2 cm^3^) once daily, preferences for numerous protein items could have evolved over time. To supplement protein intake during reduced food consumption, especially post-surgery, it is crucial to determine if subjects can regularly consume larger quantities of protein. Additionally, for a more accurate assessment of food preferences, future studies should conduct preference assessments involving the direct comparison of two or more food items. Despite its acknowledged limitations, our study represents an initial exploration into the dietary preferences of crab-eating macaques within a laboratory setting. Moving forward, it is imperative for subsequent investigations to integrate foraging activities into dietary choice assessments, thus aligning the research more closely with the natural behavior of these primates and fostering a more comprehensive understanding of their preferences.

Additional studies should be undertaken to rigorously test the applicability of the observed potential food inclination in real-world behavioral assessments. This extended research effort would contribute substantially to the practical implementation of our findings and provide a more nuanced understanding of how dietary preferences may influence the behavior of crab-eating macaques in diverse contexts.

The crab-eating macaque population is estimated to have declined by over 40% globally in the past 30 years, and it is anticipated to decrease by an additional 50% in the future [53,54]. The demand for these monkeys for biological research has increased significantly, leading to a rise in illegal activities such as forged CITES transactions involving wild-caught individuals [2]. However, there are opposing arguments. Hilborn and Smith reported that they could not find data supporting a significant decrease in the population of crab-eating macaques and suggested that they have the capacity to increase by 7–10% annually [55]. Moore et al. even went as far as to suggest that crab-eating macaques are excessively abundant and pose a threat to natural ecosystems [56]. However, it is also true that many crab-eating macaques are declining due to negative interactions with humans, and human-monkey interactions are on the rise [53,55,57]. In such a context, Gamalo et al. recommended improving animal welfare in breeding centers and striving to accommodate their needs as much as possible [2]. Furthermore, Hansen et al. highlighted the mistreatment of monkeys and advocated the initiation of conservation efforts for their well-being [53]. The present study endeavored to find ways of enhancing welfare during research in laboratory settings by making informed decisions about the provision of a variety of foods to captive monkeys.

## 5. Conclusions

The present study aimed to investigate the potential food inclination of crab-eating macaques in a laboratory setting, with a focus on identifying food items that could be effectively used in positive reinforcement training for behavioral assessments and health optimization. The results indicated a higher inclination for plant-based foods, including fruits and vegetables, as well as nuts, compared to animal-based protein-rich foods such as chicken and tuna. However, protein-rich foods other than tuna showed an increase in inclination over time. Additionally, there was a correlation between weight gain rate and inclination.

The findings emphasize the importance of considering individual variability in potential food inclination.

Despite the study’s limitations, including the absence of food-related foraging activities and the need for further investigation into real-world behavioral assessments, the findings suggest guidelines for enhancing positive reinforcement training and health optimization in crab-eating macaques. Future research incorporating natural foraging behaviors and testing the applicability of observed potential food inclinations in practical scenarios would provide additional insights into promoting the overall welfare and health of laboratory crab-eating macaques.

## Figures and Tables

**Table 1:** Nutritional value per 100 g of each of the food items presented to 12 male and 3 female crab-eating macaques to assess potential food inclination.

		Energy (Kcal)	Protein (g)	Lipids (g)	Carbohydrates (g)	Total Sugars (g)	Cholesterol (g)	Total Fatty Acids (g)
Fruits	Blueberries	48	0.55	0.09	12.57	9.96	0.00	0.08
	Shine Muskat grapes	66	0.38	0.10	17.66	15.29	0.00	0.05
	Hard persimmons	51	0.41	0.04	13.66	10.52	0.00	0.04
	Pears	45	0.29	0.04	12.24	8.30	0.00	0.04
	Mandarins	39	0.53	0.10	10.04	7.99	0.00	0.06
Vegetables	Carrots	31	1.02	0.13	7.03	6.23	0.00	0.12
	Lettuce	15	1.06	0.10	3.35	2.17	0.00	0.09
	Chinese cabbage (baechu)	15	1.25	0.04	3.20	1.75	0.00	0.04
	Paprika	24	0.77	0.20	5.95	2.79	0.00	0.12
	Cucumber	14	1.22	0.02	0.05	138.00	0.00	0.02
Proteinitems	Shrimp (dried)	319	28.50	2.00	46.00	0.00	0.00	0.00
	Japanese flying squid (dried)	352	67.80	6.90	52.00	0.00	0.00	0.00
	Chicken breast	127	28.00	0.93	0.00	0.00	74.54	0.89
	Roasted egg	142	14.20	6.90	5.70	0.00	250.4	2.4
	Tuna	216	19.00	15.00	0.00	0.00	45	0.00
Nuts	Pistachios	584	25.99	48.89	20.82	8.15	0.00	46.76
	Peanuts	567	28.50	46.24	19.91	5.18	0.00	44.23
	Walnuts (roasted)	713	14.60	68.80	11.70	2.50	0.00	67.41
	Cashews (roasted)	581	16.84	47.77	30.16	5.01	0.00	42.95
	Almonds (roasted)	594	23.45	51.29	20.49	3.91	0.00	49.01

## Data Availability

The data presented in this study are available upon request from the corresponding author.
